# Notes and News

**Published:** 1891-08-22

**Authors:** 


### Notes and News.

COLLECTED AND COMMUNICATED.

Much has been said as to the deficiency of hospital accommodation for Manchester. The same fault cannot be found with its provision for the insane. The report of the Lunacy Commissioners on the Asylum, recently issued, was of an exceptionally satisfactory nature. During the past year important additions have been made at Monsall. A new nurses' home and a new pavilion hospital, both built upon the most modern principles, are now fast approaching completion. When these are occupied there will be accommodation for 100 nurses and 354 patients.

It is difficult to take up any list of honours in the scholastic world nowadays in which women's names do not appear to the fore. In medical as in other subjects they are proving themselves well able to hold their own. The name of one lady appears in the list of candidates who have won honours in the Intermediate Examination in Medicine of London University. Miss Elizabeth Moffett, a student of the London School of Medicine for Women, is one of three candidates who have taken first-class honours in organic chemistry. Three ladies of the same school—Miss Armitage, Miss Knight, and Miss Hughes—have also passed the examination.

The *Paris Medical Journal*, in comparing the death-rate of England and Italy, supply us with a subject for reflection at a time when our attention is being especially directed to hygienic and sanitary matters. After stating that the death-rate per 1,000 in England reached 17·8 in 1889, and in Italy 27·6, it goes on to give some of the facts collected through an inquiry of a Sanitary Commission in the last-named country in 1885. The Commission stated that 6,404 Italian parishes were without drains of any kind, that in 3,636 parishes the greater number of houses were without closets, and that over an area containing nearly one-third of the population, undrinkable, indifferent, or insufficient water only was procurable.

Signor Verdi's hospital near Fiorenzuola cost in building £8,000. He added an endowment of £40,000. He is now building in Milan a hospital for poor old artists, which will cost him £20,000. The architect is Camillo Boito. Verdi will endow this hospital with a capital of £80,000. The number of aged artists admitted will be 130; they will be taken from every province in Italy, and will be most comfortably boarded and lodged. A Genoese contemporary, in recording these benefactions, records also Verdi's dislike of his charitable deeds being published. "I don't want to be reduced to end my days in a hospital," he said. "If I could manage that no one took any notice of the little good I attempt to do, I should live much more peacefully, and what little means are necessary for my own maintenance would be less threatened."

The West of France Railway has been carrying out a valuable series of experiments with various ambulance carriages on the line from Paris to Dieppe. A number of specially constructed carriages of different designs, as well as ordinary waggons with special couplings, &c., were tried. Thirty soldiers of the 131st Regiment represented the patients, who were attended and watched by Dr. Dujurdin-Beaumetz, military surgeons and physicians, officers of the general military staff, and railway officials. The train was purposely run under varying speed, now going slow, fast, and at extra-express speed, and even slight concussions were sustained, so as to represent as closely as possible the transport of wounded during war-time. Minute observations were taken; the military and medical men being well satisfied with the general result. It is probable that a report of the experiment will either be published or read before one of the scientific societies.

A correspondent writes to the *Telegraph:* A Norfolk Rector (the Rev. Thomas Berney) recently published the following extraordinary advertisement in a local paper—"Special thanksgiving services will be performed by the Rector at eleven and three o'clock for the recovery of his parish from a general though mild attack of influenza, The Hall (the Rector's residence) alone having been exempt; the epidemic being considered as a national judgment for the Papal idolatry, to be abhorred of all faithful Christians, prevailing in the Church through Jesuit traitors and their weak-minded, deluded dupes—2,000 at least—but never in his time at Bracon Ash Church. The offertory, morning and afternoon will be given to the Church Association towards the expenses of their Appeal against the Lambeth Judgment, which panders to it; and special subscriptions towards the same are very earnestly requested by the Rector, by whom they will be thankfully acknowledged. He has already received the sum of four guineas from the two Misses Berney. Were that 'Lambeth Judgment' to be confirmed, the Church of England would soon cease to be Protestant, and the blessing of the Most High, which until this influenza has so long rested upon Protestant England, would be alienated from her, and that, too, when the great and terrible war is looming."

A new use for electricity has been discovered by M. Sherer, a Frenchman. He has invented a clever but simple method of electrically doing to death gnats, flies, and similar pests, which should prove of considerable interest to the inhabitants of our colonial empire, east and west, and antipodean, as well as to denizens of Africa and certain parts of Europe. The only drawback is that he requires an electric battery giving a constant current. But as many hotels, public buildings, and private buildings in warm climates are now lighted by electricity, there can be little difficulty in setting up economical, effective, and perfectly safe death traps for aerial pests. His device is very simple. He takes a candle, lamp, or torch and places it within a cage of metallic wire gauze. This metallic gauze is connected with the poles of an electric machine, and duly charged with the electric current. The gnats, mosquitoes, flies, and wasps, fly to the light, touch the electrified metal, and are instantly killed. There is no possibility of their flying about half dead, and, as in the case with certain traps, conveying poison about the place. During the day the light can be replaced by some bait, raw meat, &c., to which the insect pests fly with alacrity and meet their doom. Those who have lived and travelled in countries troubled with the pests will be able to appreciate the simplicity of the device. The trap might probably prove effective on lawns where midges and cockchafers are a nuisance.

